# Sensitization of Common Allergens and Cosensitization Patterns Among Children in Guangzhou, China

**DOI:** 10.1155/jimr/8815583

**Published:** 2026-05-09

**Authors:** Xiaoyin Zeng, Haiyan Wang, Xiaoying Lin, Miaona Shen, Hailei Chen, Yan Huang, Qianwen Huang, Xiang Liu, Weiping Tan, Yong Liu

**Affiliations:** ^1^ Hematologic Laboratory of Pediatrics, Sun Yat-Sen Memorial Hospital, Sun Yat-Sen University, Guangzhou, 510120, China, sysu.edu.cn; ^2^ Guangdong Provincial Key Laboratory of Malignant Tumor Epigenetics and Gene Regulation, Sun Yat-Sen Memorial Hospital, Sun Yat-Sen University, Guangzhou, 510120, China, sysu.edu.cn; ^3^ Department of Pediatrics, Sun Yat-Sen Memorial Hospital, Sun Yat-Sen University, Guangzhou, 510120, China, sysu.edu.cn; ^4^ Department of Otorhinolaryngology, Sun Yat-Sen Memorial Hospital, Sun Yat-Sen University, Guangzhou, 510120, China, sysu.edu.cn

**Keywords:** allergic respiratory diseases, food allergen, inhalant allergen, logistic regression analysis, polysensitization patterns

## Abstract

**Background:**

Pediatric allergic diseases vary by region. Subtropical sensitization profiles and their clinical implications remain poorly defined.

**Method:**

In this retrospective study, specific immunoglobulin E (sIgE) levels to eight common allergens were obtained from children assessed for allergy‐associated symptoms in our department between November 1, 2017, and April 29, 2020.

**Result:**

Of the 1755 patients, *Dermatophagoides pteronyssinus* (Der p) (46.5%) and *Dermatophagoides farinae* (Der f; 45.7%) showed high rates of sensitization. More than 80% of shrimp‐sensitized children exhibited cross‐reactivity to Der p, Der f, and cockroach. After adjusting for potential confounding variables, inhalant sensitization (OR = 2.131; 95% confidence interval [CI]: 1.506–3.015; *p*  < 0.001) and polysensitization (OR = 1.480; 95% CI: 1.023–2.141; *p* = 0.038) were both associated with an increased risk of allergic airway disease.

**Conclusion:**

Our study identified the “mite–shrimp” sensitization cluster in subtropical children and establishes inhalant sensitization and polysensitization as the key risk factor in allergic respiratory diseases. These findings support the development of region‐specific and component‐resolved diagnostic strategies for pediatric allergy prevention.

## 1. Introduction

The surge in pediatric allergic diseases in China has become a prominent public health concern amidst rapid economic development and urbanization [1]. This upward trend is attributed to the complex interplay between genetic predisposition and environmental factors, with environmental exposures playing a pivotal role [2]. The intricate distribution of allergens in diverse regions, coupled with the cosensitization to multiple allergens, contributes to the complexity of allergic reactions in pediatric patients [3]. Studies indicate that a substantial proportion (60%–80%) of patients seeking specialized medical treatment for allergic respiratory diseases exhibit sensitivities to multiple allergens [4]. The development of allergies involves complex and poorly understood mechanisms. Effective treatment and prevention depend on accurately identifying the causative allergens and avoiding exposure [5].

House dust mites (HDM) are the leading aeroallergens responsible for sensitization in Asia and globally [6]. In addition, egg and milk are the most commonly identified food allergens in pediatric populations [7]. A previous study analyzing serum‐specific immunoglobulin E (sIgE) results from 39,570 patients found that only 34.6% were sensitized to a single allergen, while over 38% demonstrated sensitization to multiple (more than two) allergens [8]. Therefore, assessing the cosensitization rates among HDM, milk, egg, and other common inhalant allergens or food allergens—as well as evaluating their clinical impact—is essential. Insights into cosensitization patterns can inform strategies for allergen avoidance and guide allergen‐specific immunotherapy. However, despite its clinical relevance, limited research has explored the prevalence and implications of allergen cosensitization.

Luo et al. [3] analyzed multicenter data from 44,156 patients across seven major regions of Mainland China (2015–2018) to compare patterns of allergen‐sIgE sensitization and found that dust mites were the most common allergen. Sensitization profiles varied significantly by region (e.g., higher sensitization to dust mites and shellfish in the south and dog dander in the north), as well as by sex (higher in males), age, and season [3]. Given the significant regional variations in allergen distribution and population susceptibility across mainland China, unraveling the allergen sensitization patterns becomes imperative [9]. Taking into account epidemiological data from Guangzhou [8] and the Codex Alimentarius Commission (CAC) [10] regulations on food or ingredients that must be indicated on prepackaged food labels, we selected *Dermatophagoides pteronyssinus* (Der p), *Dermatophagoides farinae* (Der f), cockroach, dog dander, egg white, milk, wheat, and shrimp as the screening panel for suspected allergens. In this study, we conducted a retrospective analysis of allergen test results from our hospital between November 2017 and April 2020, aiming to provide data that support the characterization of regional allergen profiles and the formulation of targeted preventive and therapeutic approaches to reduce the burden of allergic diseases and enhance patient outcomes.

## 2. Methods

### 2.1. Study Subject

As a retrospective study, we included the pediatric patients (≤18 years) who underwent testing for serum allergen sIgE and exhibited clinical symptoms indicative of suspected atopic diseases, specifically allergic rhinitis (such as runny nose, sneezing, itching, or nasal obstruction), skin allergies (such as rashes, wheals, eczema, or urticaria), and allergic asthma (such as wheezing, shortness of breath, and/or cough unrelated to common cold).

A total of 1755 patients were included based on the availability of complete allergen testing records and documented clinical symptoms consistent with allergic disease. All patients were assessed at Sun Yat‐sen Memorial Hospital of Sun Yat‐sen University, between November 2017 and April 2020.The study received approval from the Institutional Ethical Committee of Sun Yat‐sen Memorial Hospital of Sun Yat‐sen University (SYSKY‐2024‐129‐01), and written informed consent was obtained from the parents of each patient.

### 2.2. Sample Collection and Measurement of Allergen sIgE

Serum samples were obtained from each patient by collecting 5 mL of venous blood. The samples were processed using gel vacuum coagulation tubes and centrifuged at 4000 rpm for 2 min. The resulting serum was then separated and prepared for allergen testing, including the following: Der p, Der f, cockroach, dog dander, egg white, milk, wheat, and shrimp.

Serum allergen‐sIgE antibodies were measured using the Phadia ImmunoCAP 250 system (Thermo Fisher Scientific, Sweden), which quantitatively detects IgE antibodies specific to individual allergens. Each allergen was assessed separately, and sensitization was defined as an sIgE level ≥0.35 kUA/L for that specific allergen. A positive test result corresponded to an sIgE level equal to or exceeding 0.35 kUA/L, classified as Class 1 or above. Based on the absolute sIgE levels, the reactivity was quantitatively classified into six categories: Class 1, ≥0.35 kUA/L to <0.70 kUA/L; Class 2, ≥0.70 kUA/L to <3.50 kUA/L; Class 3, ≥3.50 kUA/L to <17.50 kUA/L; Class 4, ≥17.50 kUA/L to <50.00 kUA/L; Class 5, ≥50.00 kUA/L to <100.00 kUA/L; Class 6, ≥100.00 kUA/L.

### 2.3. Statistical Analysis

The statistical analyses for this study were conducted with SPSS 25.0 and GraphPad Prism Software 8.0. The chi‐square test was employed to assess between‐group differences in the prevalence of sIgE positivity across sex difference and age groups. The Mann–Whitney *U* test was applied for between‐group comparisons. For the clustering analysis, we utilized the complete linkage algorithm with Euclidean distance as the distance measure. This method tends to group allergens with similar sIgE levels together. The clustering was conducted using the R package.

Spearman rank correlation analysis was employed to examine associations among the eight allergen groups, assessing the strength and direction of relationships. Radar charts were utilized to visually compare different characteristics of single or multiple subjects. Univariate and multivariable logistic regression analyses were used to investigate the risk factors for allergic respiratory disease. The logistic regression models were constructed using the enter method. Univariate analysis was first performed to identify candidate variables. Candidate variables that were significant in the univariable analysis were be consider included in the multivariable analysis. A *p*‐value <0.05 was considered statistically significant. Prior to multivariable logistic regression model inclusion, collinearity among variables was assessed using tolerance and variance inflation factors (VIFs). Multicollinearity was considered problematic if VIF values >5.

## 3. Result

### 3.1. Demographic Characteristics of the Study Population

In this study, a comprehensive analysis was conducted on a total of 1755 pediatric patients, with a mean age of 5.5 years (range 1 month to 18 years). Among the participants, 1136 (64.7%) were male, and 1185 (67.5%) were sensitized to at least one allergen. The patients were categorized into four distinct age groups: (1) infant group (0–2 years) with 409 cases; (2) preschool age group (3–5 years) with 586 cases; (3) school age group (6–11 years) with 609 cases; (4) adolescent group (12–18 years) with 151 cases. A summary of the demographic characteristics of each study group is provided in Table 1.

**Table 1:** Patient characteristics of the study cohort.

Characteristics	Total
Sex, *n* (%)	
Male	1136 (64.7)
Female	619 (35.3)
Age (mean ± SD)	5.5 ± 3.8
Allergic respiratory disease, yes/no (%)	386/1369 (22.0/78.0)
Age group, *n* (year)	
Infant (0, 2)	409 (23.3)
Preschool age (3, 5)	586 (33.4)
School age (6, 11)	609 (34.7)
Adolescent (12, 18)	151 (8.6)
Number of positive combined allergens, *n* (%)	
0	570 (32.5)
1	224 (12.8)
2	468 (26.7)
3	176 (10.0)
4	200 (11.4)
≥5	117 (6.7)

### 3.2. Allergen Sensitization Rates and Age‐Specific Distributions

Upon descending order, allergen positivity rates were determined as follows: Der p (46.5%, 95% confidence interval [CI]: 44.2%, 48.8%), Der f (45.7%, 95% CI: 43.4.2%, 48.0%), milk (29.9%, 95% CI: 27.7%, 32.0%), egg white (23.6%, 95% CI: 21.6%, 25.6%), cockroach (12.9%, 95% CI: 11.4%, 14.5%), shrimp (10.9%, 95% CI: 9.5%, 12.4%), wheat (6.2%, 95% CI: 5.1%, 7.3%), and dog dander (3.6%, 95% CI: 2.8%, 4.5%). Through chi‐square test analysis, except for wheat, the positive rates of other allergens significantly varied among different age groups (Table 2). Figure S1 illustrates the distribution and trends of allergen positivity rates across various age groups. Specifically, cockroach and shrimp induced a peak in sensitization during adolescence, while positivity rates for egg white, milk, and wheat decreased with age. Der p, Der f, and dog dander exhibited a peak in sensitization during the school‐age period in children. Furthermore, Der p and Der f were more likely to affect patients after infancy, whereas egg white and milk showed the highest prevalence of sIgE positivity during infancy. Notably, shrimp sensitization in the infant group was low (2.7%, 95% CI: 1.1%–4.3%); however, the wide CI reflects limited precision, underscoring the need for studies with larger cohorts to obtain more reliable estimates.

**Table 2:** Positive rates of allergen‐specific immunoglobulin E (sIgE) across different age groups.

Group/statistic	Prevalence of allergen positivity (%) (95% CI)							
	d1	d2	i6	e5	f1	f2	f4	f24
Total	46.5 (44.2, 48.8)	45.7 (43.4, 48.0)	12.9 (11.4, 14.5)	3.6 (2.8, 4.5)	23.6 (21.6, 25.6)	29.9 (27.7, 32.0)	6.2 (5.1, 7.3)	10.9 (9.5, 12.4)
Infant	17.6 (13.9, 21.3)	16.1 (12.6, 19.7)	1.7 (0.4, 3.0)	0.7 (−0.1, 1.6)	35.2 (30.6, 39.9)	56.0 (51.2, 60.8)	7.1 (4.6, 9.6)	2.7 (1.1, 4.3)
Preschool age	45.9 (41.9, 50.0)	45.4 (41.3, 49.4)	10.2 (7.8, 12.7)	3.1 (1.7, 4.5)	29.5 (25.8, 33.2)	33.3 (29.5, 37.1)	6.3 (4.3, 8.3)	8.5 (6.3, 10.8)
School age	62.7 (58.9, 66.6)	62.1 (58.2, 65.9)	20.0 (16.8, 23.2)	5.7 (3.9, 7.6)	14.6 (11.8, 17.4)	14.8 (12.0, 17.6)	5.9 (4.0, 7.8)	16.6 (13.6, 19.5)
Adolescent	62.6 (53.7, 69.4)	60.9 (53.1, 68.8)	25.2 (18.2, 32.2)	5.3 (1.7, 8.9)	5.3 (1.7, 8.9)	6.6 (2.6, 10.6)	4.6 (1.2, 8.0)	19.9 (13.4, 26.3)
*χ* ^2^	187.5	223.938	96.834	19.248	97.318	241.686	1.291	64.327
*p* value	<0.001 ^∗∗∗^	<0.001 ^∗∗∗^	<0.001 ^∗∗∗^	<0.001 ^∗∗∗^	<0.001 ^∗∗∗^	<0.001 ^∗∗∗^	>0.05	<0.001 ^∗∗∗^

*Note:* Data are expressed as proportion with corresponding 95% confidence intervals (95% CI). d1, Der p; d2, Der f; e5, dog dander; f1, egg white; f24, shrimp; f2, milk; f4, wheat; i6, cockroach.

^∗^ *p* < 0.05.

^∗∗^ *p* < 0.01.

^∗∗∗^ *p* < 0.001.

### 3.3. Sensitization Rates to Allergens: Sex and Seasonal Effects

**Figure 1:**
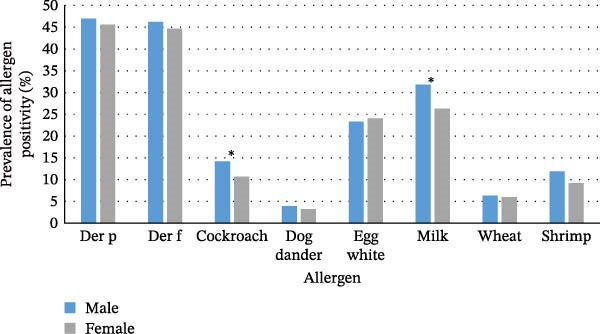
The prevalence of positive sIgE allergen detections varies between different sexes. The analysis was conducted on the entire study cohort of pediatric patients (*N* = 1755), comprising *n* = 1136 males and *n* = 619 females. Differences in the prevalence of sensitization to each allergen between males and females were evaluated using the Chi‐square test. Asterisks indicate a statistically significant difference between sexes. ( ^∗^ *p* < 0.05).

Statistically significant differences in the rate of positive sIgE detection between males and females were observed only for cockroach and milk (*χ* ^2^ = 4.384, *p* = 0.036; *χ* ^2^ = 5.673, *p* = 0.017; Figure 1). Regarding seasons, the number of people sensitized to allergens remained relatively stable throughout each season, although slightly higher sensitization to Der p, Der f, cockroach, and shrimp was observed during summer (Figure S2). As depicted in Table S1, Der p, Der f, and shrimp exhibit significant differences across the four seasons (*χ* ^2^ = 13.715, *p* = 0.003; *χ* ^2^ = 12.614, *p* = 0.006; *χ* ^2^ = 13.919, *p* = 0.003). To complement this analysis, we have added the monthly prevalence of sensitization rates for each allergen in Figure S3.

### 3.4. Spectrum of Allergen Sensitization Levels Across the Population

**Figure 2:**
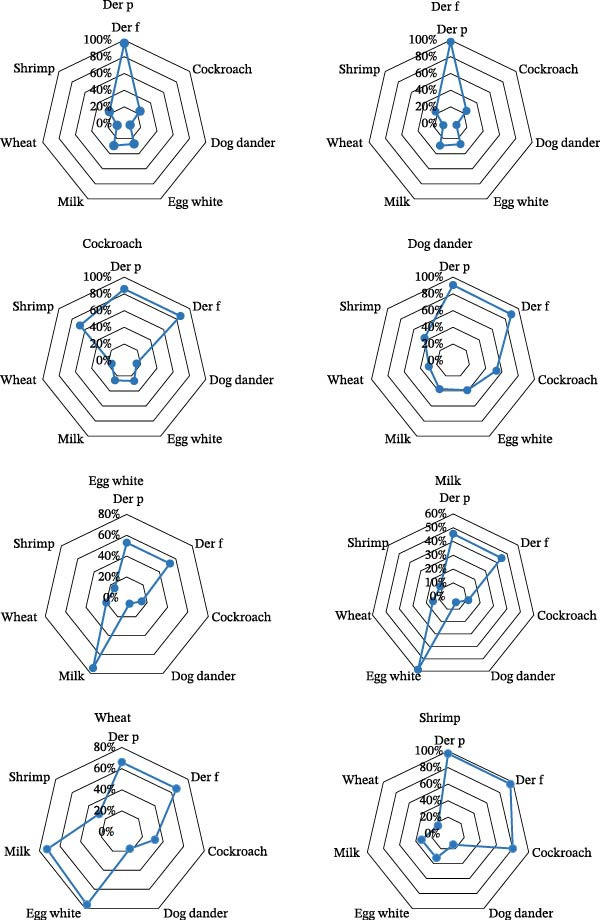
Radar chart illustrating cosensitization patterns among subjects sensitized to a single allergen. The data are presented as the percentage of co‐sensitization among patients within the overall cohort (*N* = 1755) who tested positive for the respective index allergen (Der p, Der f, cockroach, dog dander, egg white, milk, wheat, and shrimp).

Throughout the study, sensitization levels and rates to seven additional allergens where investigated among patients sensitized to a single allergen. As illustrated in Figure 2, the radar chart depicts a similar sensitization trend among patients sensitized to Der p and Der f, cockroach and shrimp, as well as egg white and milk, in comparison to the other seven allergens. Patients sensitized to Der p exhibited the highest co‐sensitization rate with Der f (96%), whereas those sensitized to Der f showed the highest cosensitization rate with Der p (98%). Predominant sensitization occurred to Der p and Der f, along with cockroach, dog dander, and shrimp (all >85%). Among patients sensitized to egg white, the most prevalent cosensitization was with milk (74%). Conversely, in individuals sensitized to milk, the most common cosensitization was to egg white (59%). Der p and Der f were the most common cosensitizing indoor allergens in this cohort of multiple sensitization children (Figure S4). When classifying sIgE reactivity in patients positive for more than one allergen simultaneously, a frequent association of high‐class sensitized responses (≥Class 3) was observed with Der p and Der f. Egg white and milk were associated with low‐class sensitized responses (Classes 1 and 2).

### 3.5. Correlation Among Different Allergens

**Figure 3:**
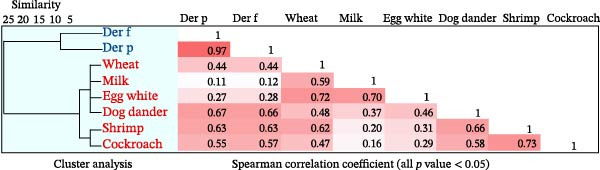
Analysis of allergen clusters and correlation among patients. The analysis encompasses the complete patient cohort (*N* = 1755). Heatmap values represent correlation coefficients (rs) between sIgE levels of the eight evaluated allergens. Correlation strength and direction were assessed using Spearman rank correlation analysis, with all displayed correlations being statistically significant (*p* < 0.05). The dendrogram illustrates hierarchical clustering of allergens based on similar sIgE levels, performed utilizing the complete linkage algorithm with Euclidean distance as the measure.

The cluster analysis results across different allergens indicated two multiple sensitization clusters: Cluster 1 includes Der p and Der f; Cluster 2 comprises wheat, egg white, milk, dog dander, cockroach, and shrimp (Figure 3). Spearman correlation analysis revealed that the correlation between Der p–sIgE and Der f–sIgE was the highest (*r*_s_ = 0.97, *p*  < 0.05), while the correlation between Der f–sIgE and milk–sIgE was the lowest (*r*_s_ = 0.11, *p*  < 0.05). Strong correlations were also observed between egg white and wheat (*r*_s_ = 0.72); egg white and milk (*r*_s_ = 0.70); dog dander and Der p (*r*_s_ = 0.67); dog dander and Der f (*r*_s_ = 0.66); dog dander and shrimp (*r*_s_ = 0.66); shrimp and cockroach (*r*_s_ = 0.73). All *p* values are below 0.05.

### 3.6. Risk for Development of Allergic Respiratory Disease

Based on clinical diagnoses, patients identified with asthma, allergic rhinitis, and atopic cough are categorized as individuals with allergic respiratory diseases in this study. Univariate logistic regression analysis revealed that age, sensitization category, and sensitization to Der p, Der f, dog dander, and milk were significant risk factors influencing the incidence of allergic respiratory disease in children (*p* < 0.05; Table 3). Notably, the winter season and milk sensitization exhibited distinct trends regarding the incidence of allergic respiratory disease. In this study, “inhalant sensitization” was defined as sensitization exclusively to inhalant allergens (either single or multiple), “food sensitization” as sensitization exclusively to food allergens (either single or multiple), and “polysensitization” as sensitization to both inhalant and food allergens. To avoid multicollinearity due to the high correlation between Der p and Der f (rs = 0.97), only Der f was included in the final multivariable regression model as a representative proxy for house dust mite sensitization. Multicollinearity among variables in the multivariable regression model was assessed using VIFs, with all values <5, indicating no significant collinearity (Table S2). Multivariate logistic regression analysis indicated that inhalant sensitization and polysensitization were independent risk factors for allergic respiratory disease (OR = 2.131, 95%CI: 1.506–3.015; OR = 1.480, 95%CI: 1.023–2.141).

**Table 3:** Univariable and multivariable logistic regression analysis of risk factors for allergic respiratory disease.

Factors	*B* coefficient	OR (95% CI)	*p* value
Univariable logistic regression analysis			
Sex	−0.135	0.874 (0.688–1.110)	0.270
Age			
Infant	—	Reference	—
Preschool	0.695	2.004 (1.379–2.911)	<0.001 ^∗∗∗^
School	1.215	3.372 (2.356–4.826)	<0.001 ^∗∗∗^
Adolescent	1.472	4.357 (2.754–6.894)	<0.001 ^∗∗∗^
Season			
Spring	—	Reference	—
Summer	−0.053	0.949 (0.699–1.288)	0.736
Autumn	−0.264	0.768 (0.542–1.089)	0.138
Winter	−0.475	0.622 (0.447–0.865)	0.005 ^∗∗^
Sensitization category	—	Reference	—
Inhalant sensitization	1.083	2.953 (2.183–3.995)	<0.001 ^∗∗∗^
Food sensitization	−0.246	0.782 (0.521–1.173)	0.234
Polysensitization	0.625	1.868 (1.364–2.557)	<0.001 ^∗∗∗^
Der p	0.013	1.013 (1.009–1.016)	<0.001 ^∗∗∗^
Der f	0.013	1.013 (1.010 −1.016)	<0.001 ^∗∗∗^
Cockroach	0.012	1.012 (0.976–1.050)	0.052
Dog dander	0.323	1.381 (1.013–1.883)	0.041 ^∗^
Egg white	−0.093	0.912 (0.804–1.033)	0.146
Milk	−0.155	0.856 (0.768–0.955)	0.005 ^∗∗^
Wheat	−0.043	0.958 (0.784–1.171)	0.675
Shrimp	0.013	1.014 (0.987–1.041)	0.32
Multivariable logistic regression analysis			
Der f	0.008	1.008 (1.004–1.011)	<0.001 ^∗∗∗^
Dog dander	0.109	1.115 (0.837–1.486)	0.457
Milk	−0.94	0.910 (0.816–1.014)	0.089
Sensitization category	—	Reference	—
Inhalant sensitization	0.757	2.131 (1.506–3.015)	<0.001 ^∗∗∗^
Food sensitization	−0.107	0.898 (0.584–1.381)	0.625
Polysensitization	0.392	1.480(1.023–2.141)	0.038 ^∗^

*Note:* “Inhalant sensitization” was defined as sensitization exclusively to inhalant allergens (either single or multiple), “food sensitization” as sensitization exclusively to food allergens (either single or multiple), and “polysensitization” as sensitization to both inhalant and food allergens.

^∗^ *p* < 0.05.

^∗∗^ *p* < 0.01.

^∗∗∗^ *p* < 0.001.

## 4. Discussion

Children’s allergic respiratory disease refers to the “four‐in‐one” treatment principle, which includes avoiding allergens, drug therapy, immunotherapy, and patient education [11]. This highlights the crucial significance of identifying the allergen. However, China is recognized as one of a nation characterized by a significant prevalence of allergies [12]. This is attributed not only to the escalating rates of various allergic disorders in recent years [13], but also to the vast expanse of its territory, which presents intricate distribution patterns of allergens across different regions. Conducting a localized allergen survey is instrumental in gathering empirical data crucial for the prevention, diagnosis, and treatment of region‐specific allergens. In this study, we presented several noteworthy aspects compared to prior research. First, we utilized the internationally recognized sIgE testing method, ImmunoCAP, to detect sensitization and analyzed the rates of sensitization to eight allergens across various ages, seasons, and sexes. Additionally, we examined the severity and cosensitization patterns among these eight allergens. Importantly, we propose a testing panel that could potentially be used to screen for allergic respiratory diseases.

As a representative subtropical coastal city, Guangzhou exhibits a HDM (Der p/Der f)–dominant sensitization pattern (positive rate >45%), which is markedly different from the pollen‐dominated profile observed in northwestern China, where mugwort and French chrysanthemum show positivity rates exceeding 48% [14]. This pattern is broadly consistent with the sensitization profile reported in the Korean population in 2025, in which Der f exhibited the highest sensitization rate (~39.9%) [15]. In contrast, the pollen sensitization pattern reported in Japan in 2023 showed that at least one allergen—Japanese cedar or cypress—had a positivity rate of 66.8% [16]. These regional disparities underscore the importance of understanding allergen distribution.

The age‐dependent patterns of allergen sensitization observed in this study—characterized by a peak in food allergen sensitization during infancy followed by a rise in inhalant allergen sensitization during school age. Similar trends have been reported in Asian settings. For example, the Singaporean GUSTO birth cohort identified an “early food‐and‐mite” sensitization trajectory (present in ~16% of children) that was strongly linked to early eczema and subsequent wheezing, reflecting the prominence of food allergen IgE in infancy [17]. By contrast, inhalant allergens predominated in later childhood. In Thailand, HDM and cockroach allergens were the most common sensitizers in children with respiratory allergy, and sensitization to these inhalants was significantly higher in older children (≥5 years) [18]. Likewise, a large Chinese pediatric allergy study found that nearly all tested aeroallergens (especially HDMs, pets, and pollens) were more prevalent in school‐aged children than in preschoolers [19].

Our analysis also revealed characteristic co‐sensitization clusters that echo molecular and epidemiologic patterns reported in Asia. Chief among these is the well‐known tropomyosin cluster: children sensitized to HDM, shrimp, and cockroach tended to be cosensitized. This likely reflects cross‐reactivity of tropomyosin, a muscle protein highly conserved across invertebrates [20]. Indeed, shrimp tropomyosin (Pen a 1) and HDM tropomyosin (Der p 10) share approximately 80% amino acid identity [20] and childhood HDM sensitization has been shown to predispose to shrimp sensitization at school age [21]. Our data mirror this link. However, Asero et al. [22] indicates the relationship is more complex than tropomyosin alone: several other shrimp and HDM allergens (for example, arginine kinase, myosin light chain, hemocyanin, paramyosin, and other high‐molecular‐weight proteins) show homologous structures and cross‐reactivity, and many shrimp‐allergic patients are also hypersensitive to HDM even when not sensitized to the three major HDM allergens (Der p 1, Der p 2, and Der p 23). Epidemiologic and clinical studies (including multicenter work) report a high prevalence of HDM reactivity among shrimp‐allergic adults and show that shrimp allergy often occurs in HDM‐allergic subjects who are not cosensitized to other airborne allergens. Comparable patterns have also been documented regionally: for example, Vietnamese allergy patients were commonly polysensitized to multiple HDM species and storage mites, with cockroach also prominent [23] and Korean population data show that >90% of individuals sensitized to seasonal or perennial allergens (birch, oak, cat dander, and dog dander) were cosensitized to HDM [15].

In our cohort, inhalant cosensitizations predominated, whereas food‐allergen clusters were less pronounced. Notably, the cosensitization rate of shrimp with HDM and cockroaches is over 85%. This rate is markedly higher than that reported in an Italian study [24], where all shrimp‐allergic patients were drawn from the HDM‐allergic group, and shrimp allergy occurred in 45 of 526 HDM‐allergic patients (9%), compared with 0% in 100 atopic controls not sensitized to HDM (*p* < 0.001). The unusually high cosensitization rate observed in Guangzhou may be partly attributable to subtropical dietary habits and high environmental humidity. Nonetheless, unique local food sensitizations do occur. For instance, Korean studies report frequent cosensitization to buckwheat and silkworm pupa in food‐allergic patients, reflecting dietary exposures [25]. Overall, subtropical Asian children appear to have relatively discrete food versus inhalant sensitization profiles, with inhalant allergens (mites and cockroaches) forming the core polysensitization cluster.

Finally, our multivariate logistic regression analysis found that polysensitization and inhalant sensitization were the primary drivers of allergic respiratory diseases in this setting. In our cohort, sensitization to inhalant allergens (particularly HDM and dog dander) was significantly associated with allergic respiratory diseases, whereas isolated food sensitization was not an independent predictor. Spearman correlation analysis showed that Der p and Der f were nearly perfectly correlated (rs = 0.97). Although cluster analysis grouped them into the same category (Cluster 1), this high correlation did not materially affect the interpretation of the multivariable model. Because such a strong correlation introduces substantial collinearity, a single proxy variable (Der f) was selected for inclusion in the multivariable regression model to preserve model stability. This selection strategy does not compromise the validity or interpretation of the study findings. Children with multiple inhalant sensitizations (e.g., to both HDM and cockroach) may carry an even higher risk of allergic respiratory diseases, underscoring the importance of considering the cumulative impact of inhalant allergens. The interrelationship between HDM and cockroach sensitization has also been highlighted by a Thai study, which reported that children with both asthma and rhinitis had a substantially higher prevalence of HDM and cockroach sensitization than those with only one disorder [18]. By contrast, milk sensitization was negatively associated with the risk of respiratory diseases (OR = 0.912), possibly reflecting the protective effect of early oral tolerance and the immunomodulatory influence of food antigen exposure as proposed by the “hygiene hypothesis” [26].

Identifying patterns of sensitization to common allergens can aid in assessing the risk and severity of allergy diseases [27–29]. Given the prevalence and relevance of these eight allergens, supporting the recommendation of the eight allergens as a potential diagnostic panel for allergic respiratory diseases is warranted. However, this recommendation requires further validation through prospective studies and alignment with existing guidelines before implementation.

Despite these valuable insights, the study has limitations, including its single‐center nature, potentially underestimating diagnostic rates for selected cases. The spatiotemporal distribution of allergen sensitization rates remains a critical area for future research, providing a more comprehensive understanding of the allergen patterns in mainland China. This would help clinicians more accurately identify triggers for allergic diseases and provide targeted treatments. Additionally, the study’s findings, despite a relatively modest sample size, align with previous research, suggesting representative subject selection.

## Funding

The authors do not have any financial concerns associated with this article.

## Disclosure

The final manuscript was reviewed and approved by all authors.

## Conflicts of Interest

The authors declare no conflicts of interest.

## Supporting Information

Additional supporting information can be found online in the Supporting Information section.

## Supporting information


**Supporting Information** Supporting material is available at The Journal of Immunology online. Figure S1: Sensitization to eight allergens across different age groups. Figure S2: Seasonal distribution of sensitization rates for each allergen. Figure S3: Monthly prevalence of sensitization rates for each allergen. Figure S4: Distribution of sIgE levels in multiallergen positivity. Table S1: Comparison of allergen positivity rates in different seasons. Table S2: Collinearity diagnostics for variables in the multivariable regression model.

## Data Availability

The data that support the findings of this study are available from the corresponding author upon reasonable request.
