# Supplementary material for: Sensitization of Common Allergens and Cosensitization Patterns Among Children in Guangzhou, China
Source: J Immunol Res. 2026 May 9;2026:8815583. doi: 10.1155/jimr/8815583 (PMC13157309; doi:10.1155/jimr/8815583)
Supplement: Supplementary file 1 — Supporting Information Supporting material is available at The Journal of Immunology online. Figure S1: Sensitization to eight allergens across different age groups. Figure S2: Seasonal distribution of sensitization rates for each allergen. Figure S3: Monthly prevalence of sensitization rates for each allergen. Figure S4: Distribution of sIgE levels in multiallergen positivity. Table S1: Comparison of allergen positivity rates in different seasons. Table S2: Collinearity diagnostics for variables in the multivariable regression model. [file JIMR-2026-8815583-s001.docx]

**Sensitization of common allergens and co-sensitization patterns among children in Guangzhou, China**

Xiaoyin Zeng^1, 2^, Haiyan Wang^2, 3^, Xiaoying Lin^1, 2^, Miaona Shen^1, 2^, Hailei Chen^1, 2^, Yan Huang^1, 2^, Qianwen Huang^1, 2^, Xiang Liu^2, 4^, Weiping Tan ^2, 3^, Yong Liu^1, 2^

**Supplementary materials**

**
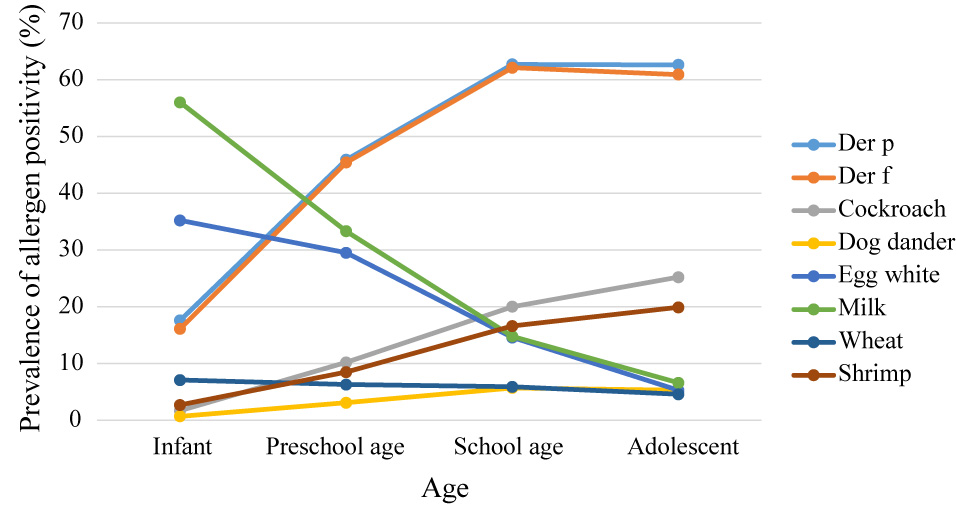
**

**Supplementary Figure S1.** Sensitization to eight allergens across different age groups. The total cohort (N=1,755) was categorized into four distinct groups: infants (0-2 years, n=409), preschool age (3-5 years, n=586), school age (6-11 years, n=609), and adolescents (12-18 years, n=151).


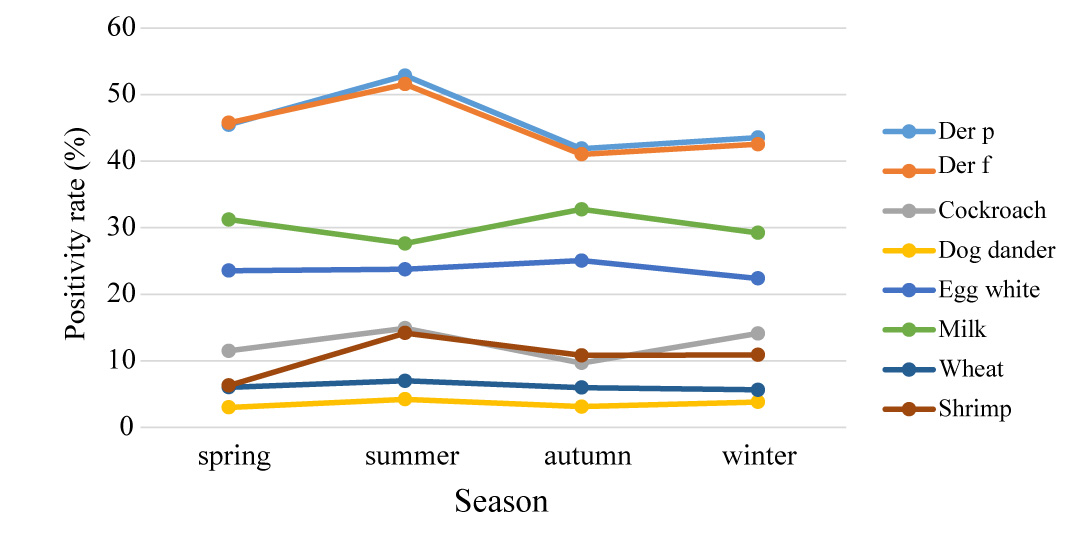


**Supplementary Figure S2.** Seasonal distribution of sensitization rates for each allergen. The analysis reflects seasonal trends across the total patient cohort (N=1,755). Seasons are defined as follows: Spring (March to May), Summer (June to August), Autumn (September to November), and Winter (December to February).

**Supplementary Figure S3.** Monthly prevalence of sensitization rates for each allergen. This analysis includes the full cohort (N=1,755).


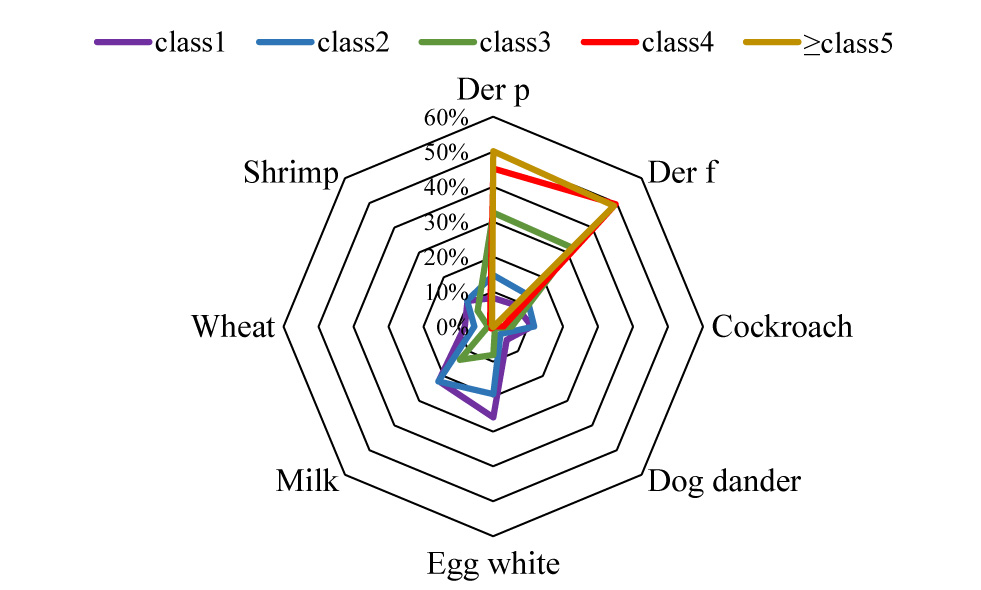


**Supplementary Figure S4.** Distribution of sIgE levels in multi-allergen positivity. The analysis illustrates the distribution of reaction severity exclusively among polysensitized patients in the cohort (those positive for ≥2 allergens simultaneously; n=961). Reactivity was quantitatively classified into categories based on sIgE levels: Class 1: ≥ 0.35 ~ 0.70 KUA/L; Class 2: ≥ 0.70 ~ 3.50 KUA/L; Class 3: ≥ 3.50 ~ 17.50 KUA/L; Class 4: ≥ 17.50 ~ 50.00 KUA/L; Class 5: ≥ 50.00 KUA/L.

**Supplementary Table S1.** Comparison of allergen positivity rates in different seasons.

| Allergens | Allergen positivity rates in different seasons, n (%) | | | |  |  |
| --- | --- | --- | --- | --- | --- | --- |
|  | Spring | Summer | Autumn | Winter | χ^2^ | P value |
| Der p | 166 (45.5) | 287 (52.9) | 147 (41.9) | 216 (43.5) | 13.715 | 0.003^**^ |
| Der f | 167 (45.8) | 280 (51.6) | 144 (41.0) | 211 (42.5) | 12.614 | 0.006^**^ |
| Cockroach | 42 (11.5) | 81 (14.9) | 34 (9.7) | 70 (14.1) | 6.455 | 0.091 |
| Dog dander | 11 (3.0) | 23 (4.2) | 11 (3.1) | 19 (3.8) | 1.263 | 0.738 |
| Egg white | 86 (23.6) | 129 (23.8) | 88 (25.1) | 111 (22.4) | 0.839 | 0.840 |
| Milk | 114 (31.2) | 150 (27.6) | 115 (32.8) | 145 (29.2) | 3.13 | 0.372 |
| Wheat | 22 (6.0) | 38 (7.0) | 21 (6.0) | 28 (5.6) | 0.903 | 0.825 |
| Shrimp | 23 (6.3) | 77 (14.2) | 38 (10.8) | 54 (10.9) | 13.919 | 0.003^**^ |

^*^*P* < 0.05; ^**^*P*< 0.01; ^***^*P* < 0.001

**Supplementary Table S2.** Collinearity diagnostics for variables in the multivariable regression model.

| Variables | Tolerance | Variance inflation factors (VIFs) |
| --- | --- | --- |
| Der f | 0.867 | 1.153 |
| Dog dander | 0.936 | 1.063 |
| Milk | 0.941 | 1.069 |
| Sensitization category | 0.855 | 1.169 |

*Note: All VIF values were < 5, indicating absence of problematic multicollinearity.
